# Water Quality Assessment and Decolourisation of Contaminated Ex-Mining Lake Water Using Bioreactor Dye-Eating Fungus (BioDeF) System: A Real Case Study

**DOI:** 10.3390/toxics12010060

**Published:** 2024-01-11

**Authors:** Zarimah Mohd Hanafiah, Ammar Radzi Azmi, Wan Abd Al Qadr Imad Wan-Mohtar, Fabrizio Olivito, Giovanni Golemme, Zul Ilham, Adi Ainurzaman Jamaludin, Nadzmin Razali, Sarina Abdul Halim-Lim, Wan Hanna Melini Wan Mohtar

**Affiliations:** 1 Department of Civil Engineering, Faculty of Engineering and Build Environment, Universiti Kebangsaan Malaysia (UKM), Bangi 43600, Malaysia; zarimahmohdhanafiah@gmail.com; 2 Functional Omics and Bioprocess Development Laboratory, Institute of Biological Sciences, Faculty of Science, Universiti Malaya, Kuala Lumpur 50603, Malaysia; ammarradzi@gmail.com; 3 Department of Environmental Engineering, University of Calabria, 87036 Rende, Italy; giovanni.golemme@unical.it; 4 Environmental Science and Management Program, Institute of Biological Sciences, Faculty of Science, Universiti Malaya, Kuala Lumpur 50603, Malaysia; ilham@um.edu.my (Z.I.); adiainurzaman@um.edu.my (A.A.J.); 5 Gamuda Land, Menara Gamuda, PJ Trade Centre, No. 8 Jalan PJU 8/8A, Bandar Damansara Perdana, Petaling Jaya 47820, Malaysia; 6 Operational and Quality Management Unit, Department of Food Technology, Faculty of Food Science and Technology, University Putra Malaysia, Serdang 43400, Malaysia

**Keywords:** *Ganoderma lucidum*, decolourisation, sustainable materials, adsorption, water quality index, mining lake

## Abstract

The environmental conditions of a lake are influenced by its type and various environmental forces such as water temperature, nutrients content, and longitude and latitude to which it is exposed. Due to population growth and development limits, former mining lakes are being converted to more lucrative land uses like those of recreational zones, agriculture, and livestock. The fungus Ganoderma lucidum has the potential to be utilised as a substitute or to perform synergistic bacteria-coupled functions in efficient contaminated lake water treatment. The purpose of this paper is to evaluate the water quality and water quality index (WQI) of an ex-mining lake named Main Lake in the Paya Indah Wetland, Selangor. Furthermore, the current work simulates the use of a Malaysian fungus in decolourising the contaminated ex-mining lake by the BioDeF system in a 300 mL jar inoculated with 10% (*v*/*v*) of pre-grown *Ganoderma lucidum* pellets for 48 h. According to the results, the lake water is low in pH (5.49 ± 0.1 on average), of a highly intense dark brownish colour (average reading of 874.67 ± 3.7 TCU), and high in iron (Fe) content (3.2422 ± 0.2533 mg/L). The water quality index of the lake was between 54.59 and 57.44, with an average value of 56.45; thus, the water was categorized as Class III, i.e., under-polluted water, according to the Malaysian Department of Environment Water Quality Index (DOE-WQI, DOE 2020). The batch bioreactor BioDeF system significantly reduced more than 90% of the water’s colour. The utilization of Ganoderma lucidum as an adsorbent material offers a variety of advantages, as it is easily available and cultivated, and it is not toxic.

## 1. Introduction

Water quality is essential for drinking water, irrigation, fish farming, recreation, and other uses that require the storage of water. Physical and chemical limnology determines water quality, which comprises all physical, chemical, and biological aspects of water that affect its wise use [1,2]. Due to over-exploitation of water supplies and poor waste disposal techniques, the quality of surface water has degraded in metropolitan areas. Watershed areas are critical for water resource conservation on both a qualitative and quantitative level [3].

Water quality degradation is related to climate change and anthropogenic sources such as agricultural and mining activities. Waste disposal from agriculture, homes, and industries is among these sources. The type of lake and its exposure to numerous environmental factors determine the lake’s environmental conditions. Lakes are a source of surface water, and their levels fluctuate throughout the year. Lakes are commonly used to satisfy the needs of a region’s population (households), industry, and agriculture. Gasim et al. [4] mentioned that surface water sources such as rivers, canals, and lakes provide nearly one-third of the world’s drinking water. As a result, surface water quality is influenced not only by natural environmental processes such as weathering, erosion, and precipitation but also by human activities such as urban, agricultural, and industrial operations [5].

Former mining lakes in Malaysia had a significant impact on economic activity. Former mining lakes are being converted to more profitable land uses such as those related to leisure areas, agriculture, and livestock due to development constraints and population increases. The scarcity of water for everyday human and home activities, particularly during the dry season, led to the search for alternative water sources. 

Physical, chemical (organic and non-organic), biological, and radiological pollutants are the five major categories of possible pollutants in mining water. According to prior research, Haan et al. [6] stated that the permissible criteria for mining water are as follows: 500–2000 mg/L for total dissolved solids (TDS), 10–100 mg/L for suspended solids (SS), 5 mg/L for biological oxygen demand (BOD), 10–100 mg/L for chemical oxygen demand (COD), 7–9.5 pH, 600–10,000 µS/cm for conductivity, and 30–600 units for colour. Ex-mining lakes in Malaysia have significant amounts of water and are expected to supply daily water needs [7]. As a result, it is vital to consider aspects connected to water analysis, particularly dangerous heavy metals that are commonly present in high amounts [8].

Heavy metals in water resources refer to a set of metals and metalloids with a density ranging from 3.5 to 7 g/cm^3^ [9]. Even at low concentrations, heavy metals such as chromium (Cr), copper (Cu), lead (Pb), and zinc (Zn) present in water resources are toxic especially to aquatic life and public health [10,11]. Souza et al. [12] state that heavy metal exposure has been related to a variety of catastrophic conditions, including developmental delays, kidney damage, various cancers, increased blood pressure and cardiovascular problems, osteoporosis, and even death in extreme cases. Nonetheless, with proper treatment and management, these abandoned mines could provide a viable source of water for local communities. As a result, more ex-mining lakes in Malaysia should be investigated and analysed to determine their potential as alternate water sources for public water supply.

Water treatment using fungal technology, also called mycoremediation, is an alternative treatment to water treatment using biological processes, specifically bacterial, due to limitations in removing all pollutants simultaneously [13]. Mycoremediation offers extensive removal of pollutants, such as heavy metals [14], antibiotics [15], dyes [16], aromatic hydrocarbons [17], organic materials, and non-organic pollutants [18], due to varying mechanistic properties. Fungi have a unique enzyme that can convert harmful pollutants into less harmful and less toxic by-products through the biodegradation or biotransformation mechanism [19], thus minimizing the discharge of toxic materials into the environment. Fungi also may act as a biosorbent, as their mycelial pellets offer ‘sponge-like’ properties, making fungi an appealing technology to explore by researchers [20]. Promising mycoremediation technology provides low costs, energy savings, and highly efficient environmental pollutant removal, and it is envisaged that the combination of fungal interactions will perform better than microbial bioreactors [13]. 

The biosorption of pollutants using fungi is an emerging technology currently used and applied through physical processes that have flexibility in operation, are highly efficient, and are low-cost [21]. A wide range of applications involve fungi being used as biosorbents, either in living cells, dead cells, or crude extracellular ligninolytic enzymes [22]. The live cells of a fungal biomass are a formation of filament assemblies called hyphae covered by a unique cell wall with a notable number of glycoproteins, chitin, lipids, and glucans [23,24]. Thus, these allow high biosorption on the fungal cell wall for wide functional groups such as hydroxyl (–OH), amine (–NH_2_), and carboxyl (–COOH) present in wastewater contaminants from industries such as textile, pesticide, and pharmaceutical industries [25]. Live fungal culture in the form of mycelium pellets offers advantages over dispersed mycelium, such as lower viscosity in reactors, easier biomass separation, and reusability after treatment. Fungal mycelium pellets also proved to be able to withstand harsh circumstances, such as fluctuations in pH environment, insufficient nutrient content, and toxic environments, thus being well-suited to application in industrial wastewater [26]. 

The current work aims to (a) explore the water quality and heavy metals content of former mining lakes; (b) compare findings with the literature on ex-mining lakes locally; and (c) investigate a potential treatment that can be used to decolourise lake water. The calculation of water quality indexes (WQI) and analysis of each parameter are included in this study to evaluate lake-water categories and potential treatments according to identified colour pollution. There is still limited research in the literature that reports the potential of using *Ganoderma lucidum* in treating coloured water, and the potential of using this strain from Malaysia has not been reported yet. Thus, our work reveals the potential of using this type of mushroom in the application of real coloured lake water. Moreover, this research aligns with Malaysian aspirations toward sustainable technologies and environmental–society–governance (ESG) directives to reduce water pollution, which has become a major global problem that could have a devastating impact on human health, ecosystems, and the economy.

## 2. Materials and Methods

### 2.1. Description of the Study Site and Sampling Locations

**Figure 1:**
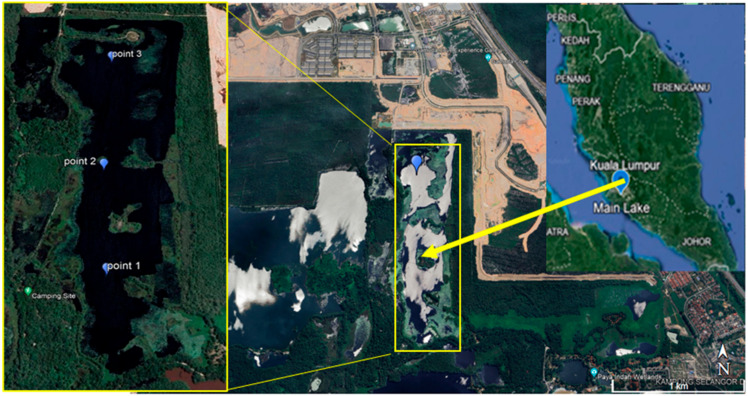
Study location at Main Lake, Paya Indah Wetland, and sampling points 1, 2, and 3.

Main Lake is located in the Paya Indah Wetland, Selangor Darul Ehsan, Malaysia as shown in Figure 1. It is a former mining lake, located in preserved land areas, and is a habitat for wildlife such as crocodiles and hippopotamuses. The Main Lake reservoir has a maximum length of 1.7 km, a maximum width of 500 m, and an average depth of 10 m. Three points were chosen to investigate the whole lake area’s water quality. Duplicate water samples were collected at each of the sampling points in the month of March 2022.

### 2.2. Water Samples and Analysis

Water quality analysis can be divided into two types of measurements: in situ measurement and laboratory measurement. Dissolved oxygen (DO) (HI-2040, Hanna Instrument, Bedfordshire, UK), electrical conductivity (EC) (CON 450, Eutech, Singapore), pH, and temperature (T) (pH450, Eutech, Singapore) were measured in situ. Transparency was evaluated using a Secchi disc. Chemical oxygen demand (COD), biochemical oxygen demand (BOD), ammonia–nitrogen (AN or NH_3_–N) and total phosphorus (TP), colour, and oil and grease (O&G) were measured using standard protocols from the manufacturer (HACH, Kuala Lumpur, Malaysia).

Heavy metals content was analysed in the laboratory using inductively coupled plasma mass spectrometry (ICPMS, ELAN 900, Perkin Elmer, Shelton, CT, USA), and microbiological content was analysed by standard methods [27]. 

Results were recorded, with the mean and standard deviation calculated using Microsoft Excel 2022.

### 2.3. Water Quality Index

The water quality index (WQI) is a set of parameters that classify surface water quality for public use, including drinking water, fishing, recreational usage, and irrigation. It is made up of six water quality indicators: BOD, DO, COD, SS, NH_3_–N, and pH. WQI classification based on parameters is summarized in Table 1.

The WQI was calculated according to the Malaysian Department of Environment Water Quality Index (DOE-WQI, DOE 2020) [28] as shown in Equation (1), where SIDO, SIBOD, SICOD, SIAN, SISS, and SIpH are subindexes for DO, BOD, COD, AN, SS, and pH, respectively.

WQI = (0.22 × SIDO) + (0.19 × SIBOD) + (0.16 × SICOD) + (0.15 × SIAN) + (0.16 × SISS) + (0.12 × SIpH)(1)

The general WQI classification is shown in Table 2, which can be classified into three categories: clean water (80–100), slightly polluted water (60–79) and polluted water (0–59).

### 2.4. Water Treatment Using Bioreactor Dye-Eating Fungus (BioDeF)

A method for converting coloured lake water into less intensely coloured water is proposed using a bioreactor dye-eating fungus (BioDeF) system. BioDeF consists of *Ganoderma lucidum* mycelial pellets in a bioreactor that act as sponges to absorb the colour and pollutants in water. *Ganoderma lucidum* was previously pre-prepared by liquid fermentation according to previous work in [29,30]. A serial batch experiment was conducted in a 300 mL jar with 10% (*v*/*v*) of the mycelial pellets in 200 mL, or the working volume of lake water. The triplicate jar was left for 48 h at room temperature and the percentage of reduction in colour was measured at the end of the batch experiment. The reduction percentage result was obtained by following Formula (2), where the percentage of the decolourisation was observed based on the difference between the average of initial absorbance (*Abs*_0_) and the average of final absorbance over time (*Abs_t_*).

(2) $$\mathrm{Decolourisation}\;\left(\%\right)=\frac{\left(Abs_{0\;}-Abs_{t}\right)}{Abs_{0}}\times 100$$

## 3. Results

### 3.1. Water Quality Parameters

#### 3.1.1. Physico-Chemical Characteristics

Tin-mining activities on land and water have caused changes to the ecosystem, both physically and chemically. Tin-mining activities produce waste such as tailing, oil, and fuel even after being abandoned for a period of time. For a clear understanding, the discussion of water quality first focuses on physico-chemical characteristics, followed by heavy metals and microbiological contents. Table 3 shows the findings of the physico-chemical characteristics of Main Lake.

For the current study, the average pH of the lake was 5.49. According to Ashraf et al. [8], the pH characteristic of abandoned tin-mining lakes tends to be acidic to neutral in the range of 4.8 to 7.2. Thus, the low pH was likely due to the solubility of chemicals and heavy metals due to post-mining activities. This finding is also in line with McCullough and Lund [31] who characterised abandoned mine pits as having a low pH and high dissolved metal concentrations resulting from acidic and metalliferous drainage (AMD). AMD is the significantly greatest problem affecting water management in the international mining industry [32].

Temperature influences the rate of biochemical and chemical reactions in a water body; higher temperatures reduce the solubility of gas and enhance the taste and odour of water. Some factors that can significantly impact the temperature of sampled water are changes in weather, time, and sampling location [4]. The average temperature reading during sampling was 29.17 °C.

Dissolved oxygen (DO) measures the amount of oxygen available in the water and identifies the ecosystem’s water quality. The higher the DO value, the better the water quality, and the lower the DO value, the worse the water quality. The mean DO value during sampling was 3.35 mg/L (40.48%) showing moderately low DO concentration in the investigated lake. This may be a consequence of the high temperature leading to high rates of decomposition of organic matter and, thus consuming more oxygen [33]. 

Electrical conductivity (EC) shows a significant relationship between water characteristics such as temperature, pH, total solids, and chemical oxygen demand (COD). Lakes with high flow or runoff clay soils tend to have higher conductivity due to the presence of ionic materials in the soils that are washed together into the water. Other than this, a failing sewage system can also influence the value of EC. For the current study, the average EC reading during sampling was 55.08 µS/cm.

For this assessment, the most apparent water quality issue was the colour. As previously mentioned, the colouration of the water body might be one of the reasons for the low DO content, with the average colour test in the lake being 875 TCU. According to the results of the samples, the average water colouration was higher than that outlined in the given NLWQS categories A and B. This scenario may limit the amount of light that enters the water body. Aquatic plants that are immersed in water require light to accomplish photosynthesis (oxygen is produced and carbon dioxide is absorbed during photosynthesis). When there is low light penetration, this leads to slow photosynthesis, finally resulting in less oxygen being generated (read as DO). The transparency of the lake water was measured using a Secchi disc; the low reading indicated that water is not transparent, and this limits light penetration into the water column and the algal growth.

The low DO concentration is accompanied by a high organic content characterizing polluted waters, as indicated by the high BOD and COD values. The average concentration of COD was 126.1 mg/L. COD is a useful indicator of organic and inorganic matter loads in surface water. Based on the NLWQS, the level of COD measured in the lake was above the limits of both categories A and B. BOD is the amount of oxygen used by microorganisms in water to decompose organic matter. The average BOD value during sampling was 8.40 mg/L above the NLWQS. A heavy downpour can also cause a high BOD value. This is because severe downpours from upstream carry a lot of mud and silt downstream. Even though this mud and silt may contain a high amount of organic matter, bacteria require a large amount of DO to stabilize the decomposition process of organic components.

The ammonia–nitrogen (NH_3_–N) and nitrate–nitrogen (NO_3_–N) concentrations in the water were 0.36 mg/L and 6.00 mg/L, respectively. The nitrogen in water is generated by heterotrophic bacteria as the primary nitrogenous product produced from the decomposition of organic matter and is readily assimilated by plants in the trophogenic zone [34]. The amount of NH_3_–N is usually low in oxygenated water in deep oligotrophic-to-mesotrophic lakes because of the utilisation of sunlight by plants in the top layer of lakes (photic zone) and nitrification forming nitrogen-oxidised forms. At relatively low dissolved oxygen concentrations, the nitrification of ammonia decreases, subsequently reducing the absorptive capacity of sediments. Under these conditions, the release of NH_3_–N from the sediments occurs [35].

The average oil and grease (O&G) content was excellent, with a value of less than 1 mg/L. Instead the total phosphorus (TP) content of the lake was 3.8 mg/L, exceeding categories A and B (NLWQS). Total suspended solids (TSS) refer to particles floating in water too large to pass through a filter, representing the presence of organic and inorganic particles in the water. TSS may also, in a way, represent the turbidity of the water. The results show that the lake’s water does not have issues with TSS and turbidity, with readings of 3.78 mg/L and 3.72 NTU, respectively.

#### 3.1.2. Heavy Metals Content

Heavy metals are generally defined as metals with a relatively high density, atomic weight or atomic number. Since heavy metals are extremely soluble in aquatic environments, they can be easily absorbed and are bioaccumulated by animals and other freshwater organisms such as aquatic and riparian vegetation, phytoplankton and zooplankton, fish and macroinvertebrates [36,37]; thus, evaluating their concentration in water is essential. Among the sources of pollution in former tin-mining lakes are heavy metals such as mercury (Hg), arsenic (As), cadmium (Cd), zinc (Zn), copper (Cu), and lead (Pb). Arsenic, mercury, cadmium and lead are some of the most dangerous heavy metals, and their poisonous nature poses a risk to the environment. They are naturally found in small amounts in water, soil, and air, but they are not required for the plant metabolism [38]. 

For the present study, based on the findings summarised in Table 4, iron (Fe) and magnesium (Mg) were the most abundant elements present in the water with concentrations of 3.24 mg/L and 1.82 mg/L, respectively. Other elements, such as sulfur (S), zinc (Zn), and manganese (Mn), were also significantly high in concentration in the water. Lead (Pb) is a natural heavy metal, abundant in the Earth’s crust. The presence of Pb does not benefit the body, even in low concentrations. Instead, it removes or replaces other metals from specific cell-binding sites, leading to various detrimental effects on human health as a growing body quickly absorbs it, and its accumulation in a developing body disrupts the natural growth of cells. After tin-mining activities, commonly generated heavy metals such as Pb, Zn, Mn, Fe, Cr, Cu, Ni, and Cd, appear dissolved in water and some of them are bound or absorbed by particulate matter, which eventually settles in the sediment bed [37].

The Main Lake water’s dark brown colour is due to inorganic and organic species; this is supported by the finding of high concentrations of heavy metals, especially those of iron (Fe) and manganese (Mn), which are suspected to be the main contributors to the water’s dark brownish colour. Toxic inorganic heavy metal complexes are usually coloured. The literature reports that heavy metals contribute to the colour of water bodies. According to Fakayode et al. [39], a light yellow aqueous colloidal P-AgNP solution changed to a dark brown colour when water samples containing Fe (II) and Mn (II) ions were added to the solution. Similarly, according to Sim et al. [40], a high concentration of soluble Fe (>0.3 mg/L) can lead to rusty staining while Mn (>0.05 mg/L) causes brownish black precipitates in plumbing fixtures. In this case, the large amounts of Fe (3.24 mg/L) and P (3.80 mg/L) suggest that insoluble FePO_4_ particles may be present in suspension.

#### 3.1.3. Microbiological Content

Other than physical and chemical properties as indicators of the quality of lake water, the contents of microorganisms such as algae, bacteria, and pathogens in lake water are also vital due to the potential for exposure to waterborne disease that is harmful to human life. The NLWQS has identified nine microbiological parameters of concern related to recreational waters (Table 5), measured by *Chlorophyll-a*, *Clostridium perfringens*, Total Coliform, *E. coli*, *Giardia* sp., *Leptospira* sp., *Cryptosporidium* sp., *Enterococci*, and *Cyanobacteria*. The results showed that the algae content (referred to as *Chlorophyll-a)* in the water was slightly high, corresponding to NLWQS categories A and B. The other microbiological contents in the water complied with the limits given in the guideline.

### 3.2. Water Quality Index Classification

The water quality index provides a detailed picture of a lake’s condition. The WQI is a rating system that measures the acceptability of water for consumption by combining the effects of numerous water quality criteria. The Main Lake water quality index was between 54.59 and 57.44, with an average value of 56.45; thus, the investigated water was categorized as Class III (Table 6). The respective subindexes of each parameter place DO in Class III, BOD in Class IV, COD in Class V, AN in Class III, TSS in Class I, and pH in Class III. Meanwhile, the water quality of the former mining lake was also analysed based on its suitability for recreational activities, as recommended by the DOE (2020). The WQI values of the study, categorized under Class III, require intensive treatment before they can be used for recreational purposes that involve water–human contact.

### 3.3. Comparison of Water Quality Parameters with the Literature

**Figure 2:**
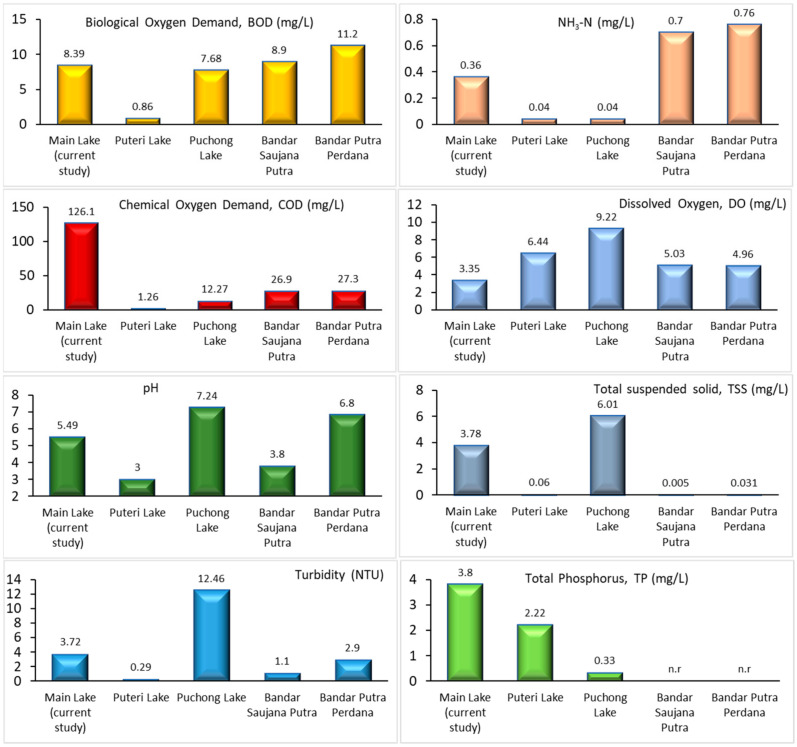
Comparison of some of physico-chemical parameters of ex-mining lake located in Selangor, Malaysia (n.r: data not reported) (Yellow bar: Biological oxygen demand, BOD; Light orange bar: Ammonia nitrogen, NH_3_–N; Red bar: Chemical oxygen demand, COD; Light blue bar: Dissolved oxygen, DO; Dark green bar: pH; Grey bar: Total suspended solid, TSS; Blue bar: Turbidity; Light green: Total phosphorus, TP).

A comparison of the water characteristics was performed based on the previously reported data of ex-mining lakes near Selangor, Malaysia, namely data from Puteri Lake [41], Puchong Lake [41], Bandar Saujana Putra [6], and Bandar Putra Perdana [6]. The water quality of man-made lakes is dependent on natural input and mining activities. As shown in Figure 2, most of the water parameters in the current study are comparable to those in previously reported work. This can be seen with BOD; Puteri Lake showed the lowest (0.86 mg/L) compared to other locations with a comparable range of 7 to 11 mg/L. For ammonia–nitrogen (NH_3_–N) content, the current study’s value was in the middle range (0.36 mg/L) compared to the highest for Bandar Saujana Putra and Bandar Putra Perdana (0.7 and 0.76 mg/L, respectively) and the lowest for Puteri Lake and Puchong Lake (0.4 mg/L). The COD of the current study was the highest (126.1 mg/L) among the considered locations (1.26 to 27.3 mg/L) and showed a high content of organic and inorganic pollutant concentrations compared to others. 

The DO concentration in Puchong Lake was highest at 9 m/L, followed by Puteri Lake, and was the lowest in the current study. The lowest DO concentration is to be expected as the high COD consumes more DO. Subsequently, the reading was low during the sampling day. The pH of fresh water may vary in alkalinity and acidity, showing that three (the current study, Puteri Lake, and Bandar Saujana Putra) of the lakes low in pH are classified as slightly acidic, and another two are in the neutral range. The suspended solids (TSS) and turbidity of the water are closely related, and Puchong Lake had the highest values for these parameters, followed by the current study and other lakes. Phosphorus content was measured in Puteri Lake and Puchong Lake but was not reported in Bandar Saujana Putra and Bandar Putra Perdana. The current study showed the highest amount of phosphorus compared to other locations. The analysis showed that the ex-mining lakes in each location differ slightly due to the geography and locations of the lakes. Apart from former mining activities, the development of residential areas, agricultural waste discharge, industrial effluent discharge, and domestic wastewater discharge are some of the main sources of pollutants in lakes. Different seasons may influence the water quality pattern of the lakes; thus, more tests may be included in future work to improve the overall data quality of ex-mining lake water, and action may be taken to improve the quality of the water.

A temporal comparison was made as part of the research work by Ahmad [42] in Main Lake in 2006/2007 as summarized in Table 7. There are no major differences that can be observed over the past 15 years in Main Lake’s water quality. As Main Lake has been part of the gazette wetland areas since 1998, natural factors (such as rainfall and nutrient content) are the major factors affecting the lake’s water quality, as indicated by BOD and COD, resulting in slightly varying concentrations over the year. The lake has also been consistently classified as WQI Class III since 2006. Apart from mining activities, Main Lake is also surrounded by 450.76 hectares of peat swamp forest areas. In general, plants in peat swamp areas decompose through the acidification process due to a lack of microbial activity, therefore causing the lake water to become acidic and dark brown in colour due to humic substances (i.e., humus and humic acid).

### 3.4. Potential Utilization of Bioreactor Dye-Eating Fungus (BioDeF)

**Figure 3:**
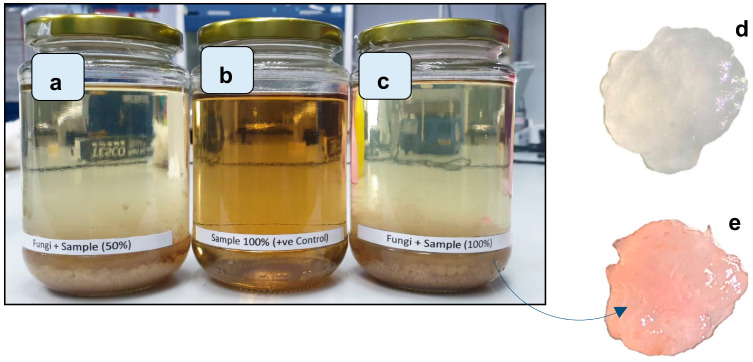
Batch bioreactor dye-eating fungus (BioDeF) after 48 h in (**a**) 50% lake water sample with 10% (*v*/*v*) fungi; (**b**) 100% lake water without fungi; and (**c**) 100% lake water sample with 10% (*v*/*v*) fungi. Under a dissecting microscope, images are shown of the biomass of *Ganoderma lucidum* (**d**) before treatment and (**e**) after treatment.

Research on the restoration of a former mining lake’s water quality needs further attention due to the lack of studies related to this type of contamination. The use of bioremediation agents is expected to be one of the technologies that can be used for water due to the advantages offered such as low cost in production [25], eco-friendliness, and non-toxic by-products [43]. In the current work, the potential of using bioreactor dye-eating fungus to reduce colour was assessed. The batch experiment is shown in Figure 3. 

The current primary work of the batch BioDeF system showed that about 96.59 ± 5.7% (Figure 3a) and 94.18 ± 3.5% (Figure 3c) of colour was significantly removed by the system. The positive control system (Figure 3b) also reduced the water slightly, by about 14.07 ± 7.8%. Furthermore, visual observation of the pellets of *Ganoderma lucidum* under a dissecting microscope (Olympus Nikon Microscope, Feasterville, PA, USA) before and after treatment showed a significant difference, as before treatment, the pellet was originally white (Figure 3d) and after treatment, the colour changed due to the adsorption process after 48 h of contact with lake water, while the fungus retained its initial structure (Figure 3e). Several studies have reported using biosorption fungal pellets in the removal of pollution from water. This includes the removal of heavy metals [14,44,45,46], phenolic compounds [18,47,48], and synthetic dyes [49,50,51] by fungal pellets, containing either a live or a dead culture. Espinosa-Ortiz et al. [14] used pellets of *Phanerochaete chrysosporium* and reduced about 88% of Zn from aqueous solutions; Bosso et al. [48] used *Anthracophyllum discolor* to remove pentachlorophenol (PCP) and achieved about >80% removal by the mechanism of adsorption; Lu et al. [49] used *Aspergillus niger* as adsorbent and achieved >98.5% removal; and similar work was conducted by Kaushik et al. [50] in reducing colour (textile dye) using fungal pellets of *Aspergillus lentulus*, reducing colour by about 90%. Previous studies show a variety of fungal pellets used as biosorbents. However, the uses of *Ganoderma lucidum* are still finite, as reported by researchers. Therefore, the current study showed that *Ganoderma lucidum* also has the potential to be used as a biosorbent to reduce colour and pollution in water.

**Figure 4:**
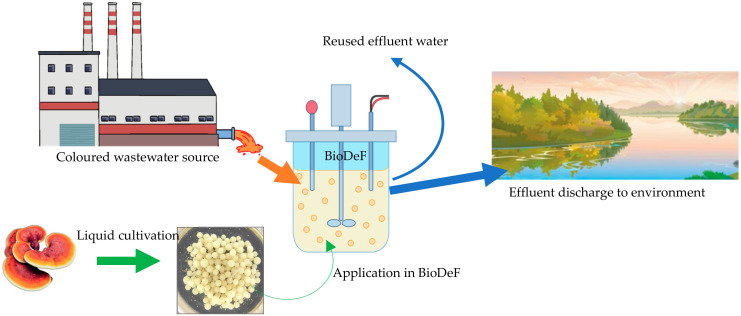
Bioreactor dye-eating fungus (BioDeF) in application of colour removal in textile effluent.

Figure 4 shows the proposed design of BioDeF in real applications of colour removal such as in textile industries and environmental water. The fungal bioreactor can be achieved by either dispersion or immobilisation of the biomass [52]. Applying pellets formed by self-immobilisation (in liquid cultivation) in BioDeF is a prominent strategy, as numerous difficulties associated with using dispersed biomasses such as insufficient mixing, clogging, difficult separation after treatment, and adhesion to reactor parts may be avoided [15,52]. Fungus treatment can be used in a continuous mode of treatment [53], thus making it viable to apply it on an industrial scale as a biosorption agent as an alternative to expensive materials like activated carbon.

It was reported that the mechanism of dye decolourisation may happen through the degradation of enzymes and biosorption. The main mechanism for dye decolourisation is degradation, as fungal enzymes such as lignin peroxidase (LiP), manganese peroxidase (MnP), and laccase degrade dyes [54]. However, different types of fungi employ different abilities of degradation enzymes and biosorption properties. In a previous work conducted by Yang et al. [55], it was reported that laccase is a major extracellular enzyme secreted by fungus *Trametes versicolor* in the decolourisation of synthetic dyes through the mechanism of biodegradation. The observation was made in parallel with the hypothesis that the decolourisation of anthraquinone-type dye is proportional to laccase enzyme activity. In another work conducted by Zhang et al. [56], it was reported that biodegradation of the fungus *Coriolus versicolor* via MnP as the main enzyme correlated to the decolourisation of cotton-bleaching effluent. LiP is the major enzyme produced by the fungus *Bjerkandera adusta* OBR105 used in the decolourisation of textile dye by Sodaneath et al. [54]. Moreover, Park et al. [57] found that there is a complex interaction of enzyme activity and biosorption mechanisms in the decolourisation of different dyes by ten types of fungal strains. Bhattacharya et al. [58] observed that the mechanism of biosorption was followed by biodegradation in the decolourisation of Congo red by fungus *Aspergillus flavus*. Previous work proved that the mechanism of dye decolourisation is a complex combination of degradation and biosorption; however, determining the exact mechanism remains a challenge as different types of fungi show different mechanisms towards different types of dyes.

## 4. Challenges, Current Perspectives, and Future Work

**Figure 5:**
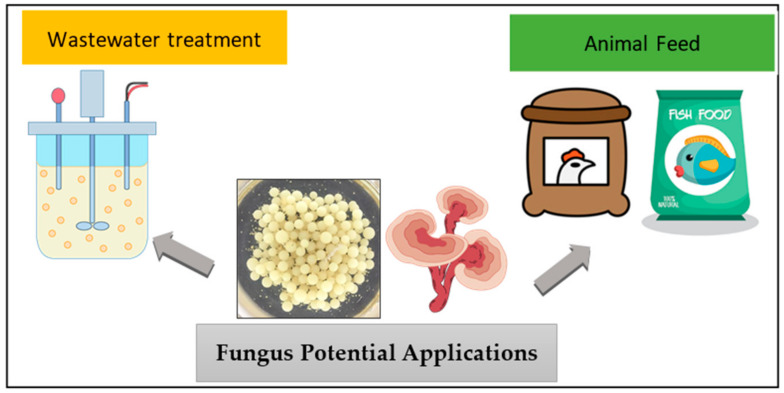
Fungi’s potential applications in wastewater treatment and animal feed industries.

Based on the previously reported biosorption and biodegradation ligninolytic enzymatic properties of fungal mycelial pellets, it cannot be denied that these little decomposers have high potential in the sorption of colour and pollutants in contaminated water. Some of the applications of fungal pellets are wastewater treatment and animal feed as shown in Figure 5. In view of wastewater treatment, fungal pellets have received attention from researchers and have been reported to be used in reducing organic and nutrient content in domestic wastewater [59,60], removing pharmaceutical products from wastewater [18,61], and pesticides [53]. Fungi can operate in a wide range of temperatures and pH values; however, the most favourable pH condition in water treatment technologies in some species of fungi is in the range of 3 to 6 [62,63] and the most favourable temperature for optimal fungal function is between 23 and 37 °C. Fungi also have antibacterial properties as they can kill bacteria in a radius of 12–21 mm, which is equivalent to the common antibiotic disc [64]. 

Fungal biomasses contain high nutritional value, are low in calories, and are high in protein and fibre content and are well known as an anti-cancer, antioxidant, and immunomodulating agent in the human diet [65]. High concentrations of branched-chain amino acids (BCAAS) in fungi can be animal-based protein sources [66]. Because of the rich properties of low levels of fats, salt, cholesterol, and calories, fungal biomasses have become a rich source of bioactive compounds that are suitable as ingredients for animal feed. Furthermore, producing edible fungal biomasses may be a more environmentally beneficial process when compared to other protein products of both animal and plant origins, leading to fewer adverse effects on the ecosystem [20]. Different species of fungal cultures may have different nutritional and bioactive compounds; thus, further investigation of the properties of fungus species is essential.

**Figure 6:**
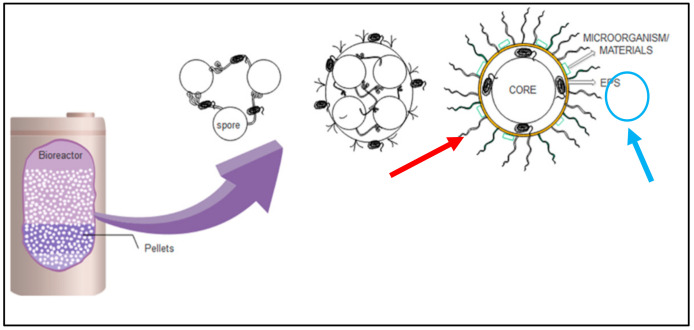
Bioreactor in wastewater or water treatment (red—materials adsorb on pellet; blue—secretion of enzyme exopolysaccharide, EPS) [67].

As discussed, various studies on the application of fungi in the removal of pollutants from water are highly promising. However, there are still challenges in designing an optimized biosorption system for pollutant removal, especially for *Ganoderma lucidum* with its limited information. The live culture of fungi needs an acclimatisation period in their surroundings before optimised sorption characteristics are achieved. Parameters such as contact time, number of fungal pellets, pH, and temperature should be studied in order to achieve optimal sorption. Furthermore, the kinetic mechanism and enzymatic characteristics of fungal treatment in batch and continuous modes of operation should be investigated. Thus, further study is important to determine limitations before the construction of upscaled bioreactor systems. Figure 6 shows the suggested bioreactor design for wastewater or water treatment and the mechanism of fungal pellets acting in the treatment process either by sorption microorganisms or materials (pollutant) (red arrow) or enzymatic degradation mechanisms through exopolysaccharide (EPS) (blue arrow). 

Incomplete mineralization of fungal degradation products is also a challenge in treatment technology. Incomplete degradation can lead to the accumulation of partially degraded products that might still be toxic or pose environmental risks. Limited study specifically reports on product transformation after studying dye treatment using various fungal species. However, a previous study reported that fungi are able to completely mineralize the dye product after treatment, thus showing that no toxic product was formed after treatment. Reviewed by Deshmukh et al. [68], white rot fungus *Schizophyllum commune* IBL-06 was able to completely decolourise the direct dye Solar Brilliant Red 80, while *C. versicolor* was able to complete degrade an azo dye, Acid Orange 7. In another study by Alvarenga et al. [69], the ability to remove the insecticide methyl parathion (MP) from seven fungal strains of *Aspergillus* and *Penicillium* taken from the sea was investigated. After 20 days of incubation, *Aspergillus sydowii* CBMAI 935 was shown to be the most effective strain in eliminating all MP. The examination of the metabolites additionally demonstrated that the development of the hazardous intermediate methyl paraoxon was dependent on the degradation pathway. Subsequently, this was completely broken down into p-nitrophenol, which resulted in a 120-fold decrease in toxicity. Despite the enzymatic degradation and biosorption capabilities of fungi, the treatment by the white rot fungus strain *Ganoderma lucidum* remains vastly unexplored and future research is needed as different cultures react differently toward different pollutants.

Fungal technologies are effective in removing various types of pollutants from wastewater; however, a lack of information in upscaling the technologies represents the main challenge in promoting them to an industrial scale. The preliminary study by Spennati et al. [70] contains baseline information that may be of use for a future real-scale application of more effective biological processes, based on the integrated use of fungi and bacteria. The work used fungi isolated from commercial tannin powder in a pilot-scale reactor using a cylindrical polypropylene vessel (volume of 1.44 m^3^) equipped with a 600 L rotating cage. The reactor was tested for 121 days, with an average 29% removal of COD and 23% removal of dissolved organic carbon (DOC) achieved. In another previous work, textile wastewater was effectively treated for an extended period of time using Fixed-Bed Reactors (FBR). In non-sterile settings, *Bjerkandera adusta* eliminated 70–80% of the colour through repeated cycles (70 days) [71]. Previous studies that tried to prolong fungal reactors in the real application of wastewater showed promising results related to fungal bioreactors. Thus, further studies of using this type of treatment technology would be useful, as local resources would benefit from wastewater treatment industries in the future.

## 5. Conclusions

Lakes are a natural resource for domestic water supply and are essential to human beings as an attractive landscape for recreational and leisure purposes. In the present study, findings revealed that most of the water quality parameters of the investigated lake are contaminated, mainly the colour properties of the lake water, due to anthropogenic activities, mainly post-mining activity, and the location of the lake being in a peat swamp forest area. Organic and inorganic waste were formed because of these conditions, and mining is polluting the reservoir. Recommendations for future work are to continue monitoring the quality of the lake water and all the related flow to the lake system of the wetland and to identify the primary pollution source. Natural treatments, such as bioremediation organisms, are the most favoured method in restoring and recovering water quality that could be adapted to the lake system in the future. Fungal biomasses, comprising one of the bioremediation technologies, offer an environmentally friendly alternative to water treatment while minimizing the release of hazardous by-product materials. In the current work, *Ganoderma lucidum* was able to remove more than 90% of the lake water’s colour after 48 h of treatment, demonstrating its high potential to be used as a biosorbent material. Despite the advantages offered by fungal technologies, designing an optimised system to achieve 100% pollutant removal is a challenge, as different species of fungal cultures may have different performance settings towards different types of dyes. Furthermore, information and data for upscaling fungal technologies to a pilot scale are still limited, representing the main challenge in promoting them to the industrial market. 

## Figures and Tables

**Table 1:** Water quality index classification for Malaysia [28].

Parameters	Unit	Class				
		I	II	III	IV	V
pH	-	>7	6–7	5–6	<5	>5
DO	mg/L	>7	5–7	3–5	1–3	<1
BOD	mg/L	<1	1–3	3–6	6–12	>12
COD	mg/L	<10	10–25	25–50	50–100	>100
SS	mg/L	<25	25–50	50–150	150–300	>300
NH_3_–N	mg/L	<0.1	0.1–0.3	0.3–0.9	0.9–2.7	>2.7
WQI	-	>92.7	76.5–92.7	51.9–76.5	31–51.9	<31

**Table 2:** General rating scale for water quality index (WQI).

Percentage of WQI (%)	Water Quality Status
80–100	Clean water
60–79	Slightly polluted water
0–59	Polluted water

**Table 3:** Physico-chemical characteristics of Main Lake water samples compared to National Lake Water Quality Standard (NLWQS) categories A and B (text with bold and underline indicates reading exceeding NLWQS).

Parameters	Mean ± SD	NLWQS	
		Category A	Category B
Temperature (°C)	29.17 ± 0.2	28	28
pH	**5.49 ± 0.1**	6.5–8.5	6.5–8.5
Colour (TCU)	**874.67 ± 3.7**	100–200	150–300
EC (µS/cm)	55.08 ± 9.1	1000	1000
Salinity (ppt)	0.04 ± 0.002	nvd	nvd
DO (mg/L)	**3.35 ± 0.5**	6.3–7.8	5.5–8.7
DO (%)	**40.48 ± 5.9**	80–100	70–110
TSS (mg/L)	3.78 ± 1.1	<100	100–500
Turbidity (NTU)	3.72 ± 0.3	40	40–170
Transparency (m)	**0.30 ± 0.02**	>0.6	>0.6
Oil and Grease (mg/L)	<1	1.5	1.5
BOD_5_ (mg/L)	**8.39 ± 0.1**	3	6
COD (mg/L)	**126.1 ± 1.3**	10	25
NH_3_–N (mg/L)	**0.36 ± 0.03**	0.1	0.3
NO_3_–N (mg/L)	6.00 ± 1.0	7	7
TP (mg/L)	**3.80 ± 0.4**	0.01	0.035

nvd: no value determined.

**Table 4:** Heavy metal concentrations in Main Lake water compared to National Lake Water Quality Standard (NLWQS) categories A and B (text with bold and underline indicates reading exceeding NLWQS).

Elements	Mean ± SD (mg/L)	NLWQS	
		Category A (mg/L)	Category B (mg/L)
Arsenic (As)	0.0022 ± 0.0001	0.05	0.10
Cadmium (Cd)	0.0001 ± 0.0001	0.002	0.002
Lead (Pb)	0.0054 ± 0.0008	0.05	0.05
Nickel (Ni)	0.0024 ± 0.0004	0.02	0.02
Iron (Fe)	**3.2422 ± 0.2533**	1.00	1.00
Magnesium (Mg)	1.8178 ± 0.0967	150	150
Manganese (Mn)	0.0673 ± 0.0020	0.10	0.10
Copper (Cu)	**0.0452 ± 0.0043**	0.02	0.02
Zinc (Zn)	0.0928 ± 0.0210	3.00	3.00
Chromium (Cr)	0.0083 ± 0.0014	0.05	0.05
Sulfur (S)	**0.3880 ± 0.1667**	0.05	0.05

**Table 5:** Microbiological contents in the Main Lake water sample compared to National Lake Water Quality Standard (NLWQS) categories A and B (text with bold and underline indicates reading exceeding NLWQS).

Microbiological	Unit	Water Sample	NLWQS	
			Category A	Category B
*Chlorophyll*-*a*	µg/L	**16.7**	10	15
*Clostridium perfringens*	Count/mL	<1	nd	nd
Total Coliform	Count/100 mL	176	5000	5000
*Total E. coli*	Count/100 mL	<1	100	600
*Giardia lamblia*	Absent/present	Absent	nd	nd
*Leptospira interrogans*	Absent/present	Absent	nd	nd
*Cryptosporidium* sp.	Absent/present	Absent	nd	nd
Enterococci	Count/100 mL	<1	33	230
Cyanobacteria	Cells/mL	3230	15,000	15,000

nd: not detected.

**Table 6:** Water quality index of Main Lake.

	**Water Quality Index (WQI)**						**WQI**	WQI quality status: Polluted water
	**SIDO**	**SIBOD**	**SICOD**	**SIAN**	**SISS**	**SIpH**		
Main Lake	35.64	67.25	9.18	68.29	95.23	74.06	56.45	
Class	III	IV	V	III	I	III	III	

**Table 7:** Temporal comparison of average water quality characteristics of Main Lake in years 2006 and 2022.

Parameters	December 2006–February 2007 [42]	March 2022 (Current)
pH	4.60 ± 0.2	5.49 ± 0.1
DO (mg/L)	2.99 ± 1.0	3.35 ± 0.5
BOD (mg/L)	5.60 ± 2.7	8.39 ± 0.1
COD (mg/L)	97.70 ± 4.8	126.1 ± 1.3
TSS (mg/L)	45.70 ± 57.4	3.78 ± 1.1
NH_3_–N (mg/)	0.50 ± 0.29	0.36 ± 0.03
Fe (mg/L)	4.28	3.2422 ± 0.2533
Mn (mg/L)	0.045	0.0673 ± 0.0020
WQI value (class)	52.9 (III)	56.45 (III)

## Data Availability

Data are available upon request.
